# Comparative Metagenomics Reveals Microbial Communities and Their Associated Functions in Two Types of Fuzhuan Brick Tea

**DOI:** 10.3389/fmicb.2021.705681

**Published:** 2021-09-16

**Authors:** Xin Wang, Gengan Du, Hong Chen, Xuejun Zeng, Bin Liu, Chunfeng Guo, Qinglin Sheng, Yahong Yuan, Tianli Yue

**Affiliations:** ^1^College of Food Science and Engineering, Northwest A&F University, Xianyang, China; ^2^Laboratory of Quality & Safety Risk Assessment for Agro-Products (Yangling), Ministry of Agriculture, Xianyang, China; ^3^College of Food Science and Technology, Northwest University, Xi’an, China

**Keywords:** Fuzhuan brick tea (FBT), microbial community, potential function, metabolic pathway, shotgun metagenomic sequencing

## Abstract

Fuzhuan brick tea (FBT) is a unique post-fermented tea product, naturally co-fermented by microorganisms, and has gained global popularity due to its potential health benefits for humans. Considerable efforts have been made toward elucidating the microbial diversity within FBT, but an understanding of the underlying FBT community interactions and functions remains poorly studied. Consequently, the microbial communities of two types of FBT, originating from Hunan and Shaanxi provinces, were investigated using comparative shotgun metagenomic sequencing and functional annotations. Metagenomic analysis indicated that two communities shared similar taxonomic and functional attributes. Two samples shared 486 genera, in which *Pseudomonas* contributed most to the abundant functions within the two samples. The carbohydrate active enzyme functions of the communities primarily comprised GH (32.92%), GT (26.8%), CEs (20.43%), and AAs (18.04%). Furthermore, the overall metabolic pathways encoded by the metagenomes were largely associated with carbohydrate and amino acid metabolism, with nine metabolic pathways that were differential between two groups including penicillin and cephalosporin biosynthesis. Significantly, a total of 35 potential probiotics were inferred, with *Pseudomonas putida* being the most abundant inferred probiotic (80%) within the FBT communities. This study provides new insights into FBT microbial communities on their potential functions and roles in FBT characteristics.

## Introduction

Fuzhuan brick tea (FBT) is a fermented tea product that has been consumed in China over 3,000 years and has since become popular in many countries (Li et al., 2018). The manufacturing of FBT comprises primary processing (proceeds from fresh tea leaves to fixing, rolling, piling fermentation, and drying) and press processing steps (screening, blending, steaming, piling fermentation, pressing, fungal fermentation, and drying) (Li et al., 2017), which requires numerous biochemical activities and microbial community successional processes that consequently produce many positive effects on human health (Liu et al., 2019). Increasing numbers of studies have confirmed the health benefits of FBT including anti-hyperlipidemia (Wang and Ho, 2009), anti-dysentery (Zhang et al., 2013), anti-obesity (Li et al., 2013), anti-hyperglycemia (Yamashita et al., 2012), and anti-oxidation (Cheng et al., 2015) effects. These health effects are closely related to the bacterial and fungal communities present within FBT that have been recently studied with culture-based or cultivation-independent methods (Tamang et al., 2016). Indeed, investigations of bacterial and fungal diversity have been intensively conducted in recent years. Nevertheless, the effects of microbial community changes on the functional diversity in FBT remain poorly understood.

The development of metagenomic methods has allowed new insights into the functions of natural microbial communities (Knight et al., 2018). Consequently, increasing attention has been paid to the integration of the microbial community genetic taxonomic and functional diversity. Concomitantly, developments over the last few decades in next-generation DNA sequencing and high-throughput computational methods have greatly enhanced our understanding of fermented food microbial communities (Arikan et al., 2020). Culture-independent taxonomic methods primarily based on high-throughput sequencing have been frequently applied to analyze fermented food microbial communities, for example, studies of wine (Johansson et al., 2021), kimchi (Kim et al., 2021), and fermented Chinese xiaoqu (Su et al., 2020). Importantly, metagenomic sequencing can identify novel species with higher resolution and better accuracy compared to traditional rRNA gene sequencing (Chen et al., 2017). Nevertheless, few studies have investigated the overall microbial community structure of FBT. Thus, efforts are needed to generate deeper insights into the underlying interactions within FBT microbial communities and their associated functions.

Hunan and Shaanxi have a long history of producing FBT in China. Thus, the objective of the present study was to comprehensively evaluate the response of microbial communities and their functions in two different types of FBT_H and FBT_S. The distribution of FBT microbial communities and their functional potentials was thus comparatively investigated by shotgun metagenomic sequencing for the first time. Detailed taxonomic and functional characteristics of the FBT communities were determined through genome assembly and subsequent comparative genomic analysis. The novel insights identified herein provide new understanding of the complex microbiomes in FBT and highlight the utility of integrated meta-omics approaches for understanding microbiomes within fermented tea product ecosystems.

## Materials and Methods

### Sample Collection

Individual FBT samples were purchased from the Yiyang Fu Cha Industry Development Co., Ltd. (Yiyang, Hunan Province, China) and the Xianyang Jingwei Fu Tea Industry Co., Ltd. (Xianyang, Shaanxi Province, China). Samples were weighed after grinding with sterile instruments. One gram of FBT was added to 10 ml of 0.1 M PBS (pH 8.0), subjected to ultrasonic extraction for 1 min and vortexed for 10 s. The above steps were repeated twice to ensure complete extraction and filtrates were collected. Subsequently, sediments were collected by centrifugating at 13,000 × *g* for 10 min, washed twice with 70% ethanol, and stored in a refrigerator at −80°C for subsequent analysis. Three replicates were set for each type of sample.

### DNA Extraction, Sequencing Library Construction, and Metagenomics Sequencing

To generate metagenomic datasets for both FBT samples, genomic DNA was extracted from 0.5 g of prepared samples using the Fast DNA SPIN Kit for Soil (MP Biomedicals, Santa Ana, CA, United States) according to the manufacturer’s protocol. The concentration and purity of DNA were quantified with a TBS-380 mini-fluorometer and NanoDrop 2000 spectrometer, respectively (Li et al., 2020). DNA was then sheared into approximately 400-bp fragments using a Covaris M220 instrument. The metagenomic libraries were subsequently prepared using the NEXTFLEX^TM^ Rapid DNA-Seq Kit (Illumina, San Diego, CA, United States). Paired-end sequencing was performed on the Illumina HiSeq 4000 platform at Majorbio Bio-Pharm Technology Co., Ltd., using a NovaSeq and HiSeq X Reagent Kits according to the manufacturer’s instructions.

### Sequence Quality Control, Contig Assembly, Gene Prediction, Taxonomic Identification, and Functional Annotation

Fastp^1^ was used to remove the adapter sequences from the 3′ and 5′ ends of paired-end Illumina reads. Low-quality reads (quality <20 or containing N bases) and short reads (<50 bp) were also removed to generate clean reads. A total of 369,511,938 high-quality sequences were obtained from two samples. Reads were then assembled *de novo* using the IDBA-UD assembler with settings of -mink 40, -maxk 97, -min_contig 300 and also mapped back to assembled contigs using Bowtie2 (Langmead and Salzberg, 2012). Unmapped reads were also assembled with MEGAHIT. The IDBA-UD and MEGAHIT assemblies were then merged and sorted into pools with fragments longer or shorter than 1,000 bp. To generate longer contigs, those <1,000 bp were assembled using Newbler (Mason et al., 2012). Contigs longer than 1,000 bp and those generated with Newbler (≥300 bp) were combined for gene prediction. Open reading frames (ORFs) were predicted using MetaGene. ORFs longer than 100 bp were translated to amino acid sequences by NCBI translation table. After sequence assembly and gene prediction, a total of 217,271 final contigs were obtained that comprised 544,374 ORFs. All protein coding genes exhibiting >90% sequence identity and 90% coverage to reference proteins were clustered using CD-HIT, and the longest sequences were chosen as representatives to construct a non-redundant gene catalog (Fu et al., 2012). The quality-filtered reads were then mapped to the representative sequences using SOAPaligner^2^ to determine gene abundances. Relative gene expression within each genome was then calculated by relativizing the expression of each ORF by the median reads per kilobase million (RPKM) value calculated across the genome (Lawson et al., 2017).

BLASTP comparison against the NR database was then used to taxonomically annotate the representative sequences using DIAMOND^3^ with an *e*-value cutoff of 1e^––5^. All unique ORFs were also functionally annotated using DIAMOND based on kyoto encyclopedia of genes and genomes (KEGG)^4^ and CAZyme^5^ databases in order to evaluate potential functions encoded by communities. KEGG modules were also used for annotating biological pathways of sequenced genomes. Overall, a non-redundant gene catalog of 121,737 genes was constructed to describe the information for genes identified in FBT samples. The SOAPaligner was used to align the high-quality reads to a non-redundant gene catalog and enumerate gene abundances.

### Statistical Analysis

Relative abundance of taxonomic groups and functional profiles was visualized using STAMP (v2.1.3). Differences in the microbial diversity were compared using ANOVA tests, with statistical significance determined by Tukey’s test (*p* < 0.05). Principal component analysis (PCA) was used to visualize the variation among the FBT microbial communities. Linear discriminant analysis effect size (LEfSe) statistical analysis was performed using the Galaxy interface.^6^ Metabolic pathways were evaluated using the online ipath2.0 platform^7^ based on KEGG annotations.

### Sequence Data Availability

The six FBT samples are available from the NCBI Sequence Read Archive. The BioProject has an accession ID of PRJNA729248. The SRA of six BioSamples is SRR14520614, SRR14520615, SRR14520616, SRR14520617, SRR14520618, and SRR14520619.^8^

## Results and Discussion

### Taxonomic Composition of the Fuzhuan Brick Tea Communities

**FIGURE 1:**
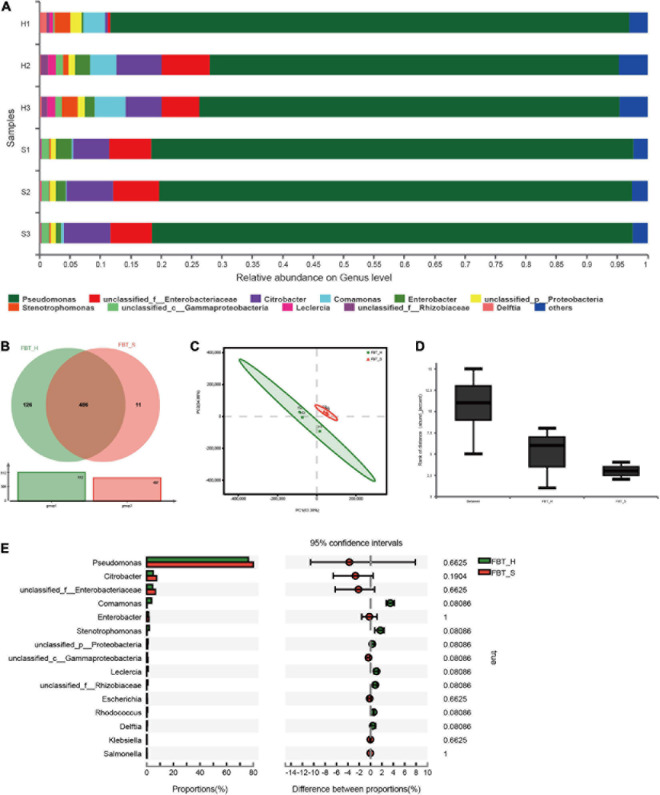
Microbial community composition at the genus level among two FBT groups. **(A)** The relative abundances of microbial populations. **(B)** Venn diagram depicting shared taxonomic groups between FBT_H and FBT_S samples. **(C)** Variation in taxonomic composition among FBT types based on PCA ordination. Different colors indicate different FBT types. The proportion of variation explained by PCA1 and 2 was 63.38 and 34.86%, respectively. FBT_H: H1–H3. FBT_S: S1–S3. **(D)** ANOSIM analysis of variation based on FBT communities. Inter-group differences are shown by the “Between” box. The “FBT_H and FBT_S” boxes show intra-group differences. **(E)** STAMP differential analysis of microbial communities between FBT_H and FBT_S samples (error bars indicate Wilcoxon rank-sum tests).

Taxonomic classification was successfully assigned to 70% of the total sequences. Approximately 99.95% of metagenomic sequences were classified as Bacteria and Archaea, with only 0.03% being classified as eukaryotic and the remaining 0.02% not being classified. The results are consistent with a previous study indicating that bacteria play indispensable roles in FBT quality with additional significant correlations observed between fungal and bacterial community compositions (Rui et al., 2019). Nearly all of the communities (98%) analyzed here were classified as Proteobacteria, with an additional small subset of community classified as Actinobacteria (<1%). Furthermore, the two FBT communities were dominated by the genus *Pseudomonas* (73.97%, 78.74%), followed by *Enterobacteriaceae* (4.83%, 7.13%), and *Citrobacter* (4.59%, 7.10%); *Comamonas* (4.35%, 0.26%), *Stenotrophomonas* (2.05%, 0.19%), *Leclercia* (1.08%, 0), and *Rhizobiaceae* (0.87%, 0) were more abundant in FBT_H than FBT_S (Figure 1A), suggesting that the difference might be due to the unique processing of FBT of the two brands not due to different regions. Moreover, it is noticed that there is greater variation in FBT_H while FBT_S replicates share more similarity. Strictly speaking, this difference is allowed in the experiment and may be caused by random error. *Pseudomonas* was clearly the most abundant taxa within FBT microbial communities, and *Pseudomonas putida* (60%) was the most abundant species within the genus (Supplementary Figure 1A). A previous study demonstrated that *P. putida* can degrade caffeine to xanthine *via* a purine-dependent metabolic pathway (i.e., N-demethylation pathway) and further alleviate the bitter and astringent taste of fermented tea (Jiang et al., 2019). Thus, *P. putida* may be an important regulator of flavor and taste profiles of fermented tea. Further metagenomic analysis indicated that the two communities shared similar taxonomic and functional attributes (Figure 1B). A total of 486 genera were shared in two samples, accounting for 78% of the total 623 genera. An additional 126 unique genera were identified in FBT_H, while 11 unique genera were only identified in FBT_S, indicating a higher level of microbial diversity in FBT_H than FBT_S.

PCA was used to evaluate variation in microbial community composition between the FBT_H and FBT_S at the genus and species level (Figure 1C and Supplementary Figure 1B). PCA clearly separated FBT_H and FBT_S with the first two components comprising 63.38 and 34.86% of the total variation, respectively. Analysis of similarities (ANOSIM) results also suggested that the intergroup differences were greater than intragroup differences, although this result did not show statistical differences (Figure 1D). The ORFs annotated against the NR database were further analyzed and the 15 most abundant genera are shown in Figure 1E. Among these, *Pseudomonas*, *Citrobacter*, and unclassified *Enterobacteriaceae* were the main taxa in two sample types, with no statistical differences in their taxonomic compositions. Previous studies showed that *Pseudomonas* sp., a potential biocontrol strain, provided beneficial protection against branch canker diseases in tea plants (Mareeswaran and Premkumar, 2020). *Pseudomonas fluorescens* also demonstrated notable antagonistic activity against fungal species, including *Fusarium oxysporum* and *Alternaria alternata* (Chakrabarti, 2009). Fu et al. (2020) suggested that *Citrobacter* sp. can degrade tea saponin (exhibits strong hemolytic and toxic properties to cold-blooded animals upon overdose) in tea seed cake, implicating a development potential within feed. In contrast, the *Enterobacteriaceae* group of *Proteobacteria* has been little studied, and their potential activities or roles in FBT require further analysis. Nevertheless, we hypothesized that the *Enterobacteriaceae* may originate from raw tea plants rather than *via* fermentation, based on previous studies (Williams et al., 2010).

*Pseudomonas putida*, unclassified *Pseudomonas*, unclassified *Enterobacteriaceae*, unclassified *Citrobacter*, and *Citrobacter freundii* were more abundant in FBT_S samples than FBT_H, while *Pseudomonas* sp. FH4, *Comamonas testosteroni*, and *P. fluorescens* were less abundant in the FBT_S samples than in FBT_H, although significant differences in abundances were again not observed (Supplementary Figure 1E). Thus, there was no significant difference in microbial community composition of FBT_H and FBT_S samples. Combined with a previous literature, it may be concluded that composition of microbial community in FBTs was mainly affected by the different fermentation stage, rather than other factors (Xu et al., 2011).

**FIGURE 2:**
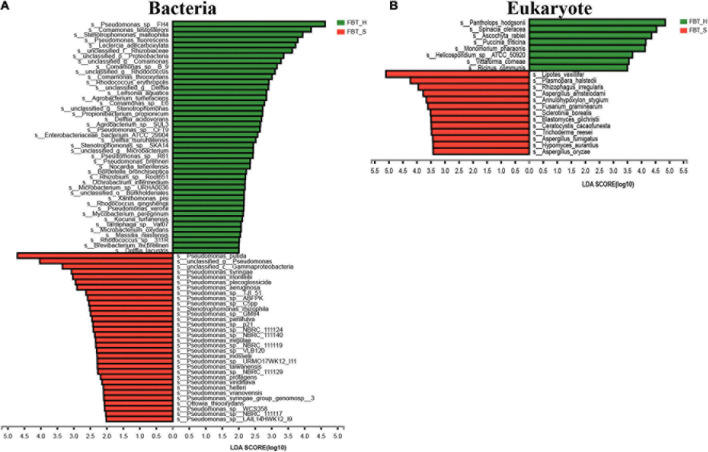
LEfSe analysis of **(A)** bacterial and **(B)** eukaryal populations that differed between FBT_H and FBT_S samples based on linear discriminant analysis (LDA) scores. Higher LDA scores indicate greater differential effects of samples on species abundances.

LEfSe analysis was then performed to confirm the differential species (species level) abundance that contributed significantly to the two FBT samples (Figure 2). A total of 76 bacteria and 21 fungi exhibited significant differences in abundances based on LEfSe analysis. *Pseudomonas* sp. FH4 and *C. testosteroni* (LDA scores > 4) were the taxa with the highest contribution scores for FBT_H enrichment. In contrast, *P. putida* and unclassified *Pseudomonas* (LDA scores > 4) were the taxa that contributed most to the FBT_S sample distinctiveness. For the eukaryotic community members, *Pantholops hodgsonii*, *Spinacia oleracea*, *Ascochyta rabiei*, *Puccinia triticina*, and *Monomorium pharao* (LDA score > 4) contributed most to FBT_H community enrichment. *Lipotes vexillifer* and *Plasmopara halstedii* (LDA scores > 4) were all identified as characteristic eukaryote species in the FBT_S samples. Numerous eukaryotic species were common to both samples and did not exhibit significant differences, including the phyla *Nematoda*, *Ascomycota*, and *Basidiomycota* (Supplementary Figures 2, 3). A previous study also reported that *Ascomycota* and *Basidaiomycota* comprised the dominant microorganisms within FBT (Zhang et al., 2013).

### KEGG Functional Analysis

**FIGURE 3:**
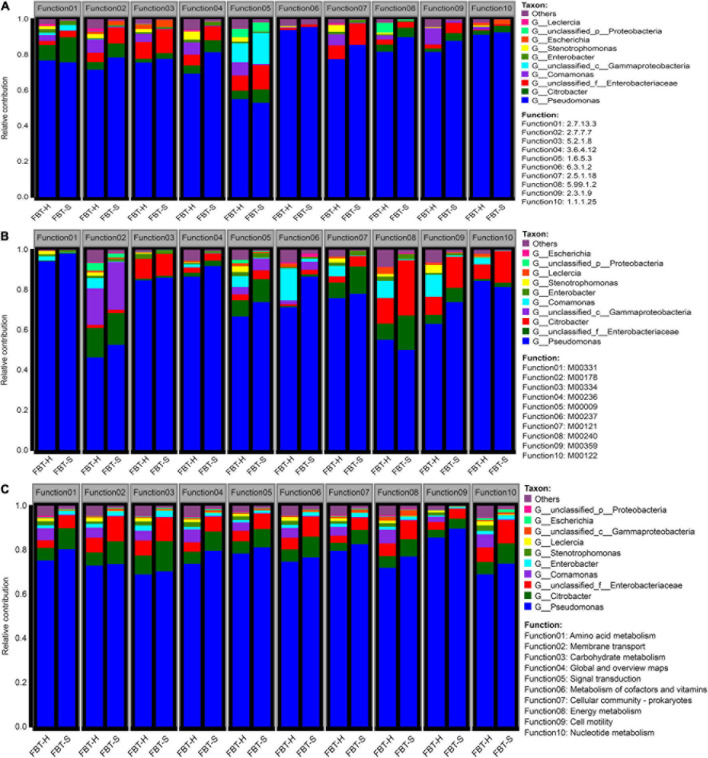
Contribution of microbial populations to **(A)** enzyme functions, **(B)** functional modules, and **(C)** KEGG pathways. Colors indicate relative abundances of different taxa. The abundance contributed by taxa = (reads of the specific taxa associated with function)/(total reads of all taxa associated with function).

The correlation coefficients of species diversity (on genus level) and enzyme functional profiles were highly consistent at 0.94 and 0.92, respectively (Supplementary Figure 5). To evaluate the functional contributions of FBT microbial populations and metabolic pathways, the functional profiles of the communities were investigated (Figure 3). Clear metabolic contributions were identified for the 10 most abundant microbial populations and specific metabolic functions. The 10 most abundant enzyme functions were contributed from *Pseudomonas* including F1: histidine kinase, F2: DNA polymerase, F3: peptidylprolyl isomerase, F4: DNA helicase, F5: NADH:ubiquinone reductase (H+-translocating), F6: glutamine synthetase, F7: glutathione transferase, F8: DNA topoisomerase, F9: acetyl-CoA C-acetyltransferase, and F10: shikimate dehydrogenase in both FBT_H and FBT_S, especially for F6 and F10 functions. Shikimate dehydrogenase mainly promoted the biosynthesis of phenylalanine, tyrosine, and tryptophan, which can synthesize important neurotransmitters and hormones involved in glucose metabolism and lipid metabolism, and then regulated the blood sugar serum lipids of the human body (Suzuki et al., 2019). It is known that glutamine synthetase can control the production of glutamine; interestingly, a literature reported that glutamine limitation can mitigate cancer (Jiang et al., 2018). The relative contributions of *Comamonas* and *Stenotrophomonas* to FBT_H functional profiles (mean = 5.08%) was much higher than FBT_S (mean = 0.43%). Conversely, the relative contributions of unclassified *Enterobacteriaceae* were lower in FBT_H (mean = 4.99%) than in FBT_S (mean = 7.72%) samples (Figure 3A). Bacterial–bacterial interactions are ubiquitous in natural environments, including in FBT. For example, the F5 function comprised genes from different bacterial taxa including unclassified *Proteobacteria*, *Stenotrophomonas*, *Enterobacter*, unclassified *Gammaproteobacteria*, *Comamonas*, unclassified *Enterobacteriaceae*, *Citrobacter*, and *Pseudomonas*. As is known to all, F5 is associated with NADH:ubiquinone reductase, a large enzyme that is the first segment of respiratory chain in most eukaryotes and many bacteria including Gammaproteobacteria (Volker et al., 2000). NADH:ubiquinone reductase is largely associated with respiration, wherein NADH can be produced during nutrient breakdown and is subsequently used to generate a proton motive force for ATP synthesis (Spero et al., 2015).

Also, microbial community and module function diversity exhibited high correlation with *R*^2^ value of 0.92 and 0.95, respectively (Supplementary Figure 5). *Pseudomonas* contributed the 10 most abundant module functions for both FBT_H and FBT_S communities (Figure 3B). In addition, considerable contributions of *Citrobacter* to the module functions of F3 (type VI secretion system), F8 (iron complex transport system), F9 (aminoacyl-tRNA biosynthesis, eukaryotes), and F10 (cobalamin biosynthesis) were also observed. The type VI secretion system is a versatile secretion mechanism involved in various functions including antibacterial activity and antagonistic inter-population competition in Gram-negative bacteria like *Citrobacter* (Gueguen and Cascales, 2013). Chen et al. (2000) reported that *Citrobacter* is a microorganism found in a compost participated in iron complex transport by producing aerobactin (a siderophore) to improve Fe acquisition. Besides, another previous study demonstrated the production of cobalamin (Vitamin B_12_) was associated with microbial fermentation in *Pseudomonas denitrificans*, *Salmonella typhimurium*, *Propionibacterium freudenreichii*, and *Propionibacterium shermanii* (Guo and Chen, 2018), suggesting that Vitamin B_12_ may exist in FBT metabolites. Thus, our results may provide new insights into cobalamin-producing bacteria taxa. Moreover, unclassified *Gammaproteobacteria* contributed the most to the abundance of function F2 (ribosome, bacteria), F5 (central carbohydrate metabolism–citrate cycle), and F6 (branched-chain amino acid transport system). Interestingly, a gene encoding a ribosome modulation factor was widely observed throughout the *Gammaproteobacteria* class organisms, but was not present in any other bacteria taxa (Ueta et al., 2013). Similarly, many *Gammaproteobacteria* utilize citric acid cycle to conserve energy in their metabolism (Assie et al., 2020). Furthermore, *Escherichia coli* belongs to the *Gammaproteobacteria* class and has been observed to use amino acid transport system to secrete threonine (Liu et al., 2012).

Considering metabolism pathway level 2 (Figure 3C), the 10 most prevalent functions were all contributed by numerous microorganisms, with more diverse species contributions for FBT_H samples compared to FBT_S. Nevertheless, *Pseudomonas* again contributed the most to functional abundances, followed by unclassified *Enterobacteriaceae* and *Citrobacter*. Interestingly, the functional contribution of *Comamonas* to FBT_H samples was much higher than FBT_S. Moreover, microbial taxa associated with the core-housekeeping functions like signal transduction (F1: two-component system), membrane transport (F2: ABC transporters), global and overview maps (F3: biosynthesis of amino acids, F4: carbon metabolism), nucleotide metabolism (F7: purine metabolism), cellular community prokaryotes (F5: bacterial secretion system, F8: biofilm formation-*Vibrio cholerae*, F9: quorum sensing), and cell motility (F6: flagellar assembly, F10: bacterial chemotaxis) were abundant metabolic categories represented in both samples (Supplementary Figure 4). The contribution of unclassified *Proteobacteria* to flagellar assembly and bacterial chemotaxis functional abundance in FBT_H samples was higher than in FBT_S samples, while the contribution of *Escherichia* to the bacterial secretion system abundance in FBT_H was much lower than in FBT_S, indicating that the functional contributions of species were related to the specific microbial environments of FBT types. Specific organisms can be associated with different metabolic functions, while the collective metabolic activities of communities can substantially modify the environment (Mazumdar et al., 2013).

**FIGURE 4:**
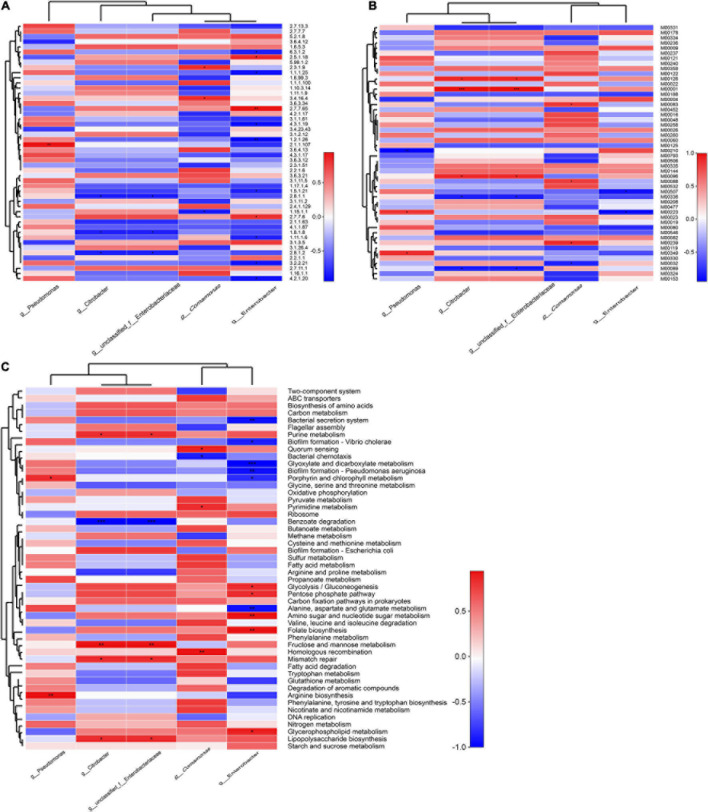
Correlation of encoded functions and the most abundant microbial taxa. Cell colors denote correlation coefficient values based on the scales to the right of the heatmaps (*p < 0.05, **p < 0.01, and ***p < 0.001). Profiles are shown for **(A)** enzyme profiles, **(B)** module profiles, and **(C)** KEGG pathway level 3.

A significant positive correlation was observed between *Pseudomonas* abundances and the enzyme function of 2.1.1.107 (uroporphyrinogen methyltransferase, *p* < 0.01), while *Enterobacter* abundances were positively correlated with 2.7.7.65 (diguanylate cyclase), but negatively correlated with 1.2.1.26 (2,5-dioxovalerate dehydrogenase, *p* < 0.01) (Figure 4A). The specific contributions of *Citrobacter* and unclassified *Enterobacteriaceae* were associated with the M00122 (cobalamin biosynthesis) (Figure 4B), consistent with the module function analysis above. Similarly, *Pseudomonas* abundances exhibited a significant positive correlation with the functional category arginine biosynthesis and porphyrin and chlorophyll metabolism (*p* < 0.01, *p* < 0.05). Chlorophyll catabolites are an indispensable part of phytochemicals and human nutrition, as Karg et al. (2021) reported that a phylloxanthobilin (a yellow chlorophyll catabolite) possesses antioxidative and anti-inflammatory activities. *Citrobacter* and unclassified *Enterobacteriaceae* abundances were also positively correlated with fructose and mannose metabolism, which may be related to the reduction of hyperglycemia by drinking FBT (*p* < 0.05) (Zhu et al., 2021); *Comamonas* abundances were also closely associated with the functions of homologous recombination, pyrimidine metabolism, and quorum sensing. In contrast, *Enterobacter* abundances were more associated with the metabolism pathways of glycerophospholipid metabolism (Figure 4C), which may participate in inhibiting fat accumulation and avoiding fatty liver produced (Wu et al., 2016).

### CAZy Functional Analysis

**FIGURE 5:**
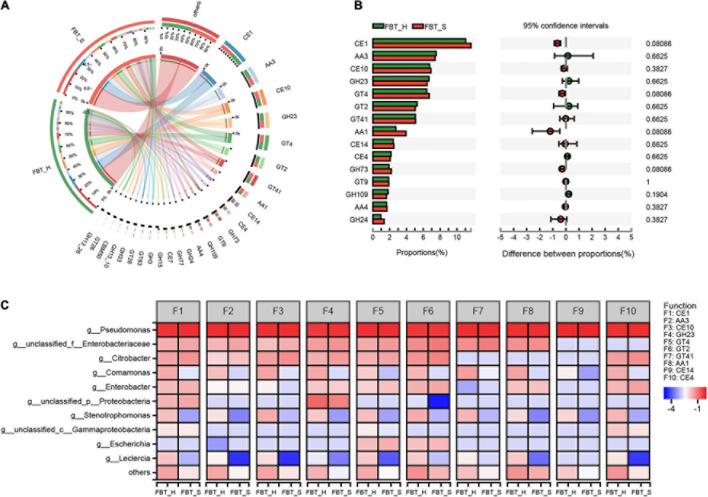
Distribution of CAZyme database functions. **(A)** The overall distribution of CAZyme among FBT samples. The length of bars for each FBT on the outer ring represents the percentage of CAZymes within each FBT sample. **(B)** Comparison of CAZyme functions between FBT_H and FBT_S samples. The data were visualized using STAMP (error bars represent Wilcoxon rank-sum test). **(C)** The contribution of species to CAZyme functional profiles. AA, auxiliary activities; CBM, carbohydrate-binding modules; CE, carbohydrate esterases; GH, glycoside hydrolases; GT, glycosyl transferases; PL, polysaccharide lyases.

A total of 6,339,740 reads were mapped to 203 CAZymes among all metagenomes (Figure 5). The carbohydrate active enzymes (CAZy) functional distributions were shown as follows: class glycoside hydrolases (GH) exhibited a functional abundance of 32.92%; glycosyl transferases (GT), 26.8%; carbohydrate esterases (CE), 20.43%; and auxiliary activities (AA), 18.04% (Supplementary Figure 5). Among these, the abundance of function CE1 (the abundance > 11%) was highest, followed by AA3, CE10, GH23, GT4, GT2, and GT41, and no significant differences were observed among the two samples (Figures 5A,B). Thus, the species contributions to CAZy functional profiles were consistent across both sample groups. Notably, GH are associated with the oxidation, conversion, or degradation of phenolic compounds (Zhao et al., 2019). A previous study also reported that a thermo-stable GH, α-rhamnosidase, may be present in FBT communities that contributed to produce glycosidically bound volatiles such as benzyl alcohol, 2-phenylethanol, methyl salicylate, linalool, geraniol, coumarin, and damascenone (Fang et al., 2019). In addition, increases in GT expression levels have been proven, which is beneficial for metabolizing starch and cellulose to glucose, thereby providing energy to populations tolerating fluoride stress (Yang et al., 2020). Moreover, Habrylo et al. (2018) reported that some CEs exhibit optimal activity at pH 6–8 and 40°C, which renders them suitable for both acidic and alkaline applications, such as coffee and tea fermentations. Similarly, most AA in FBT reflected the adaptations of microbial populations to tea substrate (Kanokratana et al., 2018).

The contributions of *Comamonas*, *Stenotrophomonas*, and *Leclercia* to each of the CAZy functions were higher in FBT_H samples than FBT_S (Figure 5C). Additionally, AA3_2 and GH103 were key characteristic CAZy functions in FBT_H samples, while AA1 was a key CAZy function for FBT_S samples based on LDA scores (Supplementary Figure 5). An obvious positive correlation was observed between *Comamonas* and the abundances of function CE4, reflecting that *Comamonas* harbors and contributes to the abundances of CE4 family enzymes including chitin deacetylase (EC 3.5.1.41), chitooligosaccharide deacetylase (EC 3.5.1.-), peptidoglycan GlcNAc deacetylase (EC 3.5.1.-) and peptidoglycan N-acetylmuramic acid deacetylase (EC 3.5.1.-). However, some significant negative correlations were observed including the CE1 function and *Comamonas* abundances (Spearman *R* = −0.94, *p* < 0.01), and CE10 and GT41 functions and *Enterobacter* abundances (Spearman *R* = 0.94, *p* < 0.01).

### Differential Metabolic Pathways

**FIGURE 6:**
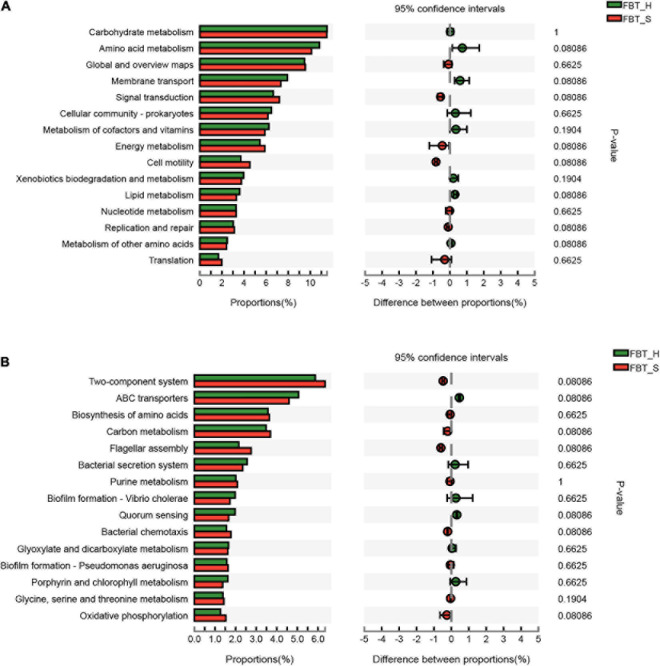
Comparison of KEGG pathway level functions between FBT type communities. **(A)** KEGG pathway level 2 and **(B)** KEGG pathway level 3.

**FIGURE 7:**
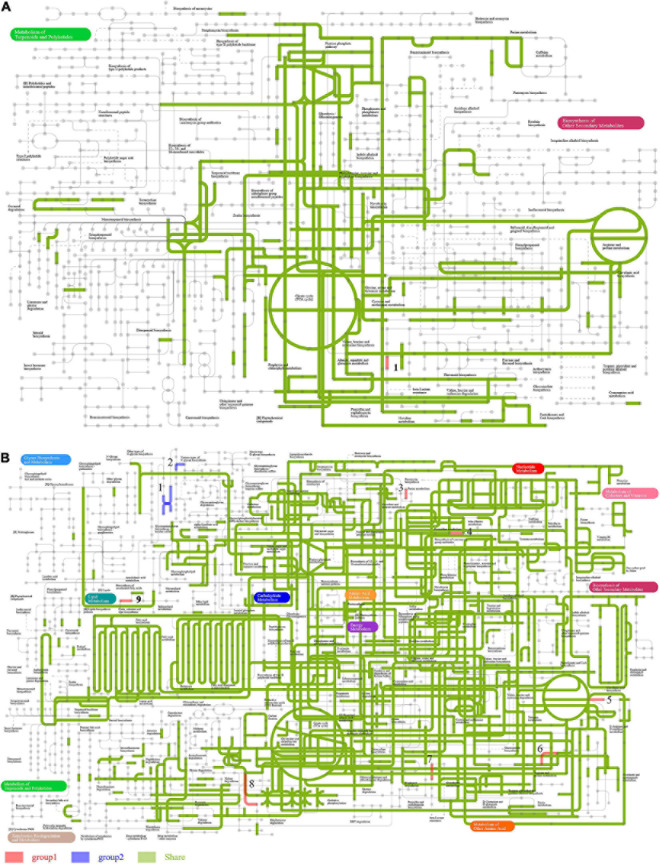
Comparison of ipath metabolic pathways between FBT-type communities. Red and blue lines indicate unique metabolic pathways for FBT_H and FBT_S communities, respectively, while green lines indicate shared metabolic pathways among the two samples. **(A)** Biosynthesis of secondary metabolites and overall **(B)** metabolic pathways.

Seven level 2 KEGG pathways were overrepresented in FBT_H samples relative to FBT_S, while six were less represented in FBT_H samples compared to FBT_S. The most represented metabolic pathways comprised carbohydrate and amino acid metabolism for both samples and their abundances were not significantly difference (Figure 6A). In addition, the two-component system (ko02020) and ABC transporters (ko02010) were the most abundant level 3 KEGG metabolism pathways. A total of 255 and 287 enzymes were primarily affiliated with the ko02020 and ko02010 pathways, respectively, all of which did not exhibit significant differences between FBT_H or FBT_S samples (Figure 6B and Supplementary Tables 4, 5). Comparative analysis of secondary metabolite biosynthesis pathways between FBT_H and FBT_S was conducted using the ipath platform, with one differential pathway (penicillin and cephalosporin biosynthesis) being observed only in FBT_H samples. This pathway is primarily involved in the coordinated transformation of two metabolites: isopenicillin N (C05557) and penicillin N (C06564) (Figure 7A). Cephalosporins and penicillins are the most frequently used β-lactam antibiotics for treating human infections globally. The primary organisms used for industrial production of these antibiotics are *Acremonium chrysogenum*, *Penicillium chrysogenum*, *Aspergillus nidulans*, and the Gram-positive bacterium *Streptomyces lipmanii* (Terfehr et al., 2017). Isopenicillin N is the bioactive intermediate of the penicillin biosynthesis pathway (Ziemons et al., 2017). Furthermore, the gene pa4191 of *Pseudomonas aeruginosa* has provisionally been annotated as a member of the isopenicillin N synthase family that mediates a key step in penicillin biosynthesis (Zhang et al., 2019). In brief, penicillin is biosynthesized by several eukaryotic and bacterial species. Metagenomic analyses showed here that several FBT community strains were involved in penicillin and cephalosporin biosynthesis including *Agrobacterium*, *Comamonas*, *Delftia*, *Leclercia*, *Microbacterium*, *Ochrobactrum*, *Paraburkholderia*, *Pseudomonas*, *Stenotrophomonas*, and unclassified *Rhizobiaceae*.

Further analysis of the global overview map of metabolic pathways provided additional insights into the central metabolisms of two FBT community populations (Figure 7B). A total of nine metabolic pathways were observed that were differentially abundant among two FBT types. Pathways I and II were related to glycan biosynthesis and only observed in the FBT_S community, while other seven central metabolic pathways (III–IX) were only present in FBT_H samples, namely, III, ascorbate and aldarate metabolism; IV, purine metabolism and pyrimidine metabolism; V, glycine, serine, and threonine metabolism, and arginine and proline metabolism; VI, cysteine and methionine metabolism; VII, penicillin and cephalosporin biosynthesis; VIII, ethylbenzene degradation; and IX, sphingolipid metabolism. Based on the above results, it is possible that greater abundances of tea polysaccharides may be produced in FBT_S type teas that contribute to reducing the risk of type 2 diabetes and obesity (Chen et al., 2019). In addition, some abundant microbial populations participated in amino acid metabolism activity (e.g., with glycine, serine, threonine, arginine, proline, cysteine, and methionine substrates) that can improve the taste of tea (Chen et al., 2020). The metabolic pathway of ethylbenzene degradation in FBT_H samples contributed to the regulation of fermented tea aroma (Zhu et al., 2018). Moreover, several microbial populations within FBT samples were also associated with the regulation of ascorbate and aldarate metabolism, nucleotide, and sphingolipid metabolism.

### Probiotic Analysis

A total of 35 probiotics were predicted (annotated against probio database) within two FBT sample groups. Among these, nine were only annotated in FBT_H samples, namely, *Vibrio alginolyticus*, *Lactobacillus helveticus*, *Serratia liquefaciens*, *Lysinibacillus fusiformis*, *Lactobacillus brevis*, *Lactobacillus crispatus*, *Lactobacillus kefiranofaciens*, *Bifidobacterium catenulatum*, and *Pantoea agglomerans*; and four were only annotated in FBT_S communities, namely, *Bacillus thuringiensis*, *Clostridium butyricum*, *Bacillus licheniformis*, and *Acinetobacter calcoaceticus* (Table 1). Obviously, *P. putida* was the most abundant inferred probiotic with 80% proportions in both sample groups, which can use caffeine as its sole source of carbon and nitrogen, rendering it a potential candidate for bio-decaffeination, the production of caffeine derivatives, and environmental remediation of caffeine (Ma et al., 2018). It also has been used to biodegrade agrochemicals like the triazole fungicide propiconazole (Sarkar et al., 2010). Thus, *P. putida* played a key role in ensuring the safety of tea products.

**TABLE 1:** Probiotic composition between FBT_H and FBT_S based on probio database.

**Probiotics only in FBT_H**	**Probiotics only in FBT_S**	**Common probiotics in FBT_H and FBT_S**
*Vibrio alginolyticus*	*Bacillus thuringiensis*	*Arthrobacter globiformis*
*Lactobacillus helveticus*	*Clostridium butyricum*	*Lactobacillus rhamnosus*
*Serratia liquefaciens*	*Bacillus licheniformis*	*Bacillus circulans*
*Lysinibacillus fusiformis*	*Acinetobacter calcoaceticus*	*Bradyrhizobium pachyrhizi*
*Lactobacillus brevis*		*Kluyvera ascorbata*
*Lactobacillus crispatus*		*Janthinobacterium lividum*
*Lactobacillus kefiranofaciens*		*Burkholderia cepacia*
*Burkholderia cepacia*		*Bradyrhizobium japonicum*
*Pantoea agglomerans*		*Azotobacter chroococcum*
		*Azospirillum brasilense*
		*Micrococcus luteus*
		*Propionibacterium freudenreichii*
		*Propionibacterium thoenii*
		*Oxalobacter formigenes*
		*Pseudomonas corrugata*
		*Aeromonas sobria*
		*Pseudomonas chlororaphis*
		*Pseudomonas fluorescens*
		*Delftia acidovorans*
		*Pseudomonas putida*
		*Pseudomonas sp.*
		*Pantoea dispersa*

**FIGURE 8:**
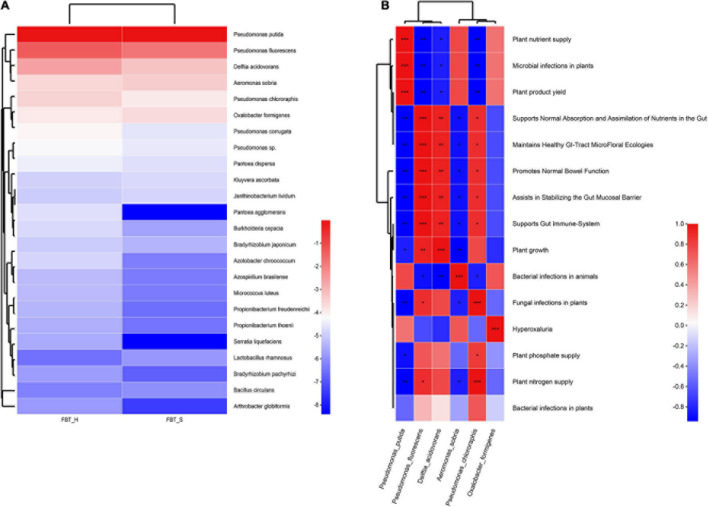
Probiotic analysis for FBT communities against probio database. **(A)** The relative abundances of inferred probiotic in FBT communities. **(B)** Correlation between probiotic and functional abundances (*p < 0.05, **p < 0.01, and ***p < 0.001). Cell colors indicate the correlation coefficients based on the scale to the right.

Additionally, the abundances of other potential probiotics in FBT_H were higher than FBT_S, except for *P. putida* and *P. fluorescens*. Thus, potential probiotic effects may be higher in FBT_H (Figure 8A). Furthermore, some significant positive correlations were observed between annotated probiotics and functions (*p* < 0.001), including the fact that *P. putida* abundances are associated with the effects of plant nutrient supplies, microbial infections in plants, and plant product yields, while the abundances of *P. fluorescens* are closely related to the normal absorption and assimilation of nutrients in intestines, the maintenance of healthy gastrointestinal tract microfloral ecologies, the promotion of normal bowel functions, assistance in stabilizing gut mucosal barrier, and supporting gut immune system. Furthermore, *Delftia acidovorans* is highly associated with plant growth effects, *Aeromonas sobria* is highly associated with bacterial infections in animals, *Pseudomonas chlororaphis* is closely related to the effects of fungal infections in plants and plant nitrogen supplies, and *Oxalobacter formigenes* is highly associated with hyperoxaluria effects (Figure 8B).

Metagenomic analysis of FBT microbial communities here provided novel insights into the huge probiotic flora of FBT. Overall, numerous previously reported probiotic fungi including *Eurotium cristatum* and *Aspergillus cristatus* (Kang et al., 2019; Rui et al., 2019) were identified from FBT, while several predicted bacteria were also identified in FBT using culture-dependent methods and 16s RNA sequencing including *P. fluorescens*, *P. putida*, *Lactobacillus rhamnosus*, *L. kefiranofaciens*, and *B. licheniformis* (Supplementary Figure 6 and Supplementary Table 6).

## Conclusion

The potential interactions of microbial populations and their associated functions were evaluated in two types of FBT using metagenomics for the first time. Our results showed that microbial community composition was largely similar among two types of FBT samples, with no significant differences. However, diversity in FBT_H (612 genera) was higher than FBT_S (497 genera). *Pseudomonas* contributed most to the abundant functional groups in both FBT_H and FBT_S. Moreover, the carbohydrate enzyme functional distribution of microbial communities in FBT exhibited highly abundant functional potential for GHs (32.92%), GTs (26.8%), CEs (20.43%), and AAs (18.04%). Carbohydrate and amino acid metabolism were the primary and key metabolic pathways in two FBT samples. Notably, *Pseudomonas* contributed the most to enzyme function, module function, and pathway level 2. A significant positive correlation was observed between *Pseudomonas* abundances and the enzyme function of 2.1.1.107 (uroporphyrinogen methyltransferase, *p* < 0.01). In addition, considerable contributions of *Citrobacter* to the module functions of F3 (type VI secretion system), F8 (iron complex transport system), F9 (aminoacyl-tRNA biosynthesis, eukaryotes), and F10 (cobalamin biosynthesis) were also observed. The specific contributions of *Citrobacter* and unclassified *Enterobacteriaceae* were associated with the M00122 (cobalamin biosynthesis). Differently, nine metabolic pathways exhibited differential abundances between two types of communities. A total of 35 probiotics were present in FBT samples, with nine being unique to FBT_H and four being unique to FBT_S communities. Furthermore, *P. putida* was the most abundant inferred probiotic (80%) among two samples. This study provided new and detailed insights into the taxonomic composition and potential function of FBT communities (including carbohydrate and metabolic functions), especially for the potential probiotics in FBT communities.

## Data Availability Statement

The datasets presented in this study can be found in online repositories. The names of the repository/repositories and accession number(s) can be found below: www.ncbi.nlm.nih.gov/, PRJNA729248.

## Author Contributions

XW prepared the manuscript, did the literature survey, and designed the figures. YY, TY, and GD revised the manuscript. HC and XZ performed some of the experiments. BL, CG, and QS revised some of the mistakes in grammar. All authors read and approved the manuscript.

## Conflict of Interest

The authors declare that the research was conducted in the absence of any commercial or financial relationships that could be construed as a potential conflict of interest.

## Publisher’s Note

All claims expressed in this article are solely those of the authors and do not necessarily represent those of their affiliated organizations, or those of the publisher, the editors and the reviewers. Any product that may be evaluated in this article, or claim that may be made by its manufacturer, is not guaranteed or endorsed by the publisher.

## Footnotes

1
https://github.com/OpenGene/fastp

2
https://github.com/aquaskyline/SOAPdenovo2

3
http://ab.inf.uni-tuebingen.de/software/diamond/

4
http://kobas.cbi.pku.edu.cn/home.do

5
http://www.cazy.org/

6 http://huttenhower.sph.harvard.edu/galaxy/root?tool_id=lefse_upload, α<0.05, LDA score>2.

7
http://pathways.embl.de

8
https://dataview.ncbi.nlm.nih.gov/?search=SUB9613785&archive=bioproject

## Supplementary Material

Supplementary Figure 1 Microbial community compositions among FBT samples. **(A)** Relative abundances of microbial community. **(B)** Variation in genus-level microbial composition within FBT samples. The proportion of variation explained by PCA1 and 2 was 80.08 and 19.15%, respectively. FBT_H: H1–H3. FBT_S: S1–S3. **(C)** Hierarchical clustering of genus-level taxonomic profiles. **(D)** Hierarchical clustering analysis of species-level profiles. **(E)** Comparison of species groups between FBT_H and FBT_S samples.

Supplementary Figure 2 LEfSe analysis of bacterial communities among FBT samples. Red nodes indicate taxa are significantly enriched in FBT_S samples, while green nodes indicate taxa are significantly enriched in FBT_H samples. Yellow nodes indicate the lack of significant effect when comparing FBT_H and FBT_S samples.

Supplementary Figure 3 LEfSe analysis of eukaryotic communities among FBT samples. Red nodes indicate taxa are significantly enriched in FBT_S samples, while green nodes indicate taxa are significantly enriched in FBT_H samples. Yellow nodes indicated the lack of significant effect when comparing FBT_H and FBT_S samples.

Supplementary Figure 4 Species and KEGG pathway level 3 contribution analysis.

Supplementary Figure 5 Distribution of CAZyme functions among FBT samples. **(A)** α diversity between microbial genus-levels and CAZyme functions. **(B)** β diversity between genus-levels and CAZyme functions. **(C)** Differences in CAZyme function between FBT_H and FBT_S samples. Higher LDA scores indicate greater effects of sample type on functional abundances. **(D)** Correlational analysis between species and CAZyme functions (^∗^*p* < 0.05, ^∗∗^*p* < 0.01, and ^∗∗∗^*p* < 0.001). **(E)** Distribution of CAZyme functions.

Supplementary Figure 6 The colony morphology of 5 potential probiotics.

Supplementary Table 1 Biosynthesis of secondary metabolite pathways enriched in FBT_H samples.

Supplementary Table 2 Metabolic pathways enriched in FBT_S samples.

Supplementary Table 3 Metabolic pathways enriched in FBT_H samples.

Supplementary Table 4 ko02010 ABC transporter annotations in FBT samples.

Supplementary Table 5 ko02020 two-component system annotations in FBT samples.
