# Supplementary material for: Comparative Metagenomics Reveals Microbial Communities and Their Associated Functions in Two Types of Fuzhuan Brick Tea
Source: Front Microbiol. 2021 Sep 16;12:705681. doi: 10.3389/fmicb.2021.705681 (PMC8481837; doi:10.3389/fmicb.2021.705681)
Supplement: Supplementary Figure 1 — Microbial community compositions among FBT samples. (A) Relative abundances of microbial community. (B) Variation in genus-level microbial composition within FBT samples. The proportion of variation explained by PCA1 and 2 was 80.08 and 19.15%, respectively. FBT_H: H1–H3. FBT_S: S1–S3. (C) Hierarchical clustering of genus-level taxonomic profiles. (D) Hierarchical clustering analysis of species-level profiles. (E) Comparison of species groups between FBT_H and FBT_S samples. [file Data_Sheet_1.ZIP › Table S1-3.docx]

**Table S1. Differential biosynthesis of secondary metabolities only in FBT_H.**

| 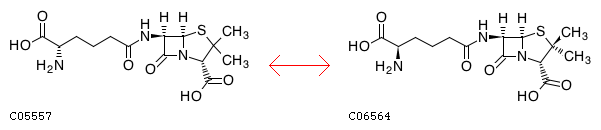 | |
| --- | --- |
| 2 compunds | C05557, Isopenicillin N; C06564, Penicillin N |
| 1 pathway | 00311, Penicillin and cephalosporin biosynthesis |
| 1 reaction | R04147 |
| 1 enzyme | 5.1.1.17, Isopenicillin-N epimerase |

**Table S2. Differential metabolic pathway only in FBT_S.**

| **1** Glycan biosynthesis | | **2** Glycan biosynthesis | |
| --- | --- | --- | --- |
| 4 compounds | C03021, Protein asparagine; C00621, Dolichol diphosphate; G00008, Glycan; G00009, Glycan. | 3 compounds | G00012, Glycan; G00011, Glycan; G10694, Glycan. |
| 3 pathways | 00510, N-Glycan biosynthesis; 00513, High-mannose type N-glycan biosynthesis; 04141, N-Glycan biosynthesis. | 3 pathways | 00510, N-Glycan biosynthesis; 00513, High-mannose type N-glycan biosynthesis; 04141, N-Glycan biosynthesis. |
| 1 reaction | R05976 | 1 reaction | R06722 |
| 1 module | M00072, N-glycosylation by oligosaccharyltransferas | 2 modules | M00073, N-glycan precursor trimming; M00074, N-glycan biosynthesis, high-mannose type |
| 2 enzymes | 2.4.1.119, Glycosyltransferases | 1 enzeyme | 3.2.1.113,mannosyl-oligosaccharide 1,2-alpha-mannosidase; |

**Table S3. Differential metabolic pathway only in FBT_H.**

| **Metabolic pathway** | **Compound synthesis** |
| --- | --- |
| **3** Ascorbate and aldarate metabolism | 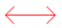C00545, L-Arabinonate C01114, L-Arabonolactone |
| **4** Purine metabolism & Pyrimidine metabolism | 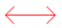C00475, Cytidine C00380, Cytosine |
| **5** Glycine,serine and threonine metabolism & Arginine and proline metabolism | 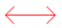C00062, L-Arginine C00581, Guanidinoacetate |
| **6** Cysteine and methionine metabolism | 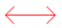C04188, S-Methyl-5-thio-D-ribose 1-phosphate  C00170, Methylthioadenosine |
| **7** Penicillin and cephalosporin biosynthesis | 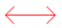C05557, Isopenicillin N C06564, Penicillin N |
| **8** Ethylbenzene degradation | 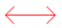C00596, 2-Hydroxy-2,4-pentadienoate  C07123, 2-Hydroxy-6-oxo-octa-2,4-dienoate |
| **9** Sphingolipid metabolism | 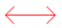C02934, 3-Dehydrosphinganin C00836, Sphinganine |
