# Supplementary material for: Comparative Metagenomics Reveals Microbial Communities and Their Associated Functions in Two Types of Fuzhuan Brick Tea
Source: Front Microbiol. 2021 Sep 16;12:705681. doi: 10.3389/fmicb.2021.705681 (PMC8481837; doi:10.3389/fmicb.2021.705681)
Supplement: Supplementary Figure 1 — Microbial community compositions among FBT samples. (A) Relative abundances of microbial community. (B) Variation in genus-level microbial composition within FBT samples. The proportion of variation explained by PCA1 and 2 was 80.08 and 19.15%, respectively. FBT_H: H1–H3. FBT_S: S1–S3. (C) Hierarchical clustering of genus-level taxonomic profiles. (D) Hierarchical clustering analysis of species-level profiles. (E) Comparison of species groups between FBT_H and FBT_S samples. [file Data_Sheet_1.ZIP › Table S6.docx]

Table S6. 16s RNA sequencing of five potential probiotics.

| Strain | Source | Total score | %Identity | Query length% |
| --- | --- | --- | --- | --- |
| *Pseudomonas fluorescens* | FBT_H & FBT_S | 2603 | 99.65 | 99 |
| *Pseudomonas putida* | FBT_H & FBT_S | 2595 | 99.58 | 99 |
| *Lactobacillus rhamnosus* | FBT_H & FBT_S | 2680 | 99.86 | 99 |
| *Lactobacillus kefiranofaciens* | FBT_H | 2684 | 99.66 | 99 |
| *Bacillus licheniformis* | FBT_S | 2084 | 99.05 | 99 |
