# Supplementary material for: Comparative Metagenomics Reveals Microbial Communities and Their Associated Functions in Two Types of Fuzhuan Brick Tea
Source: Front Microbiol. 2021 Sep 16;12:705681. doi: 10.3389/fmicb.2021.705681 (PMC8481837; doi:10.3389/fmicb.2021.705681)
Supplement: Supplementary Figure 1 — Microbial community compositions among FBT samples. (A) Relative abundances of microbial community. (B) Variation in genus-level microbial composition within FBT samples. The proportion of variation explained by PCA1 and 2 was 80.08 and 19.15%, respectively. FBT_H: H1–H3. FBT_S: S1–S3. (C) Hierarchical clustering of genus-level taxonomic profiles. (D) Hierarchical clustering analysis of species-level profiles. (E) Comparison of species groups between FBT_H and FBT_S samples. [file Data_Sheet_1.ZIP › The preparation steps of FBT.docx]

**The preparation steps of FBT**

The manufacturing process for FBT is divided into primary processing and press processing steps. Primary processing can be broken down into the following steps: fresh tea leaves→fixing→rolling→piling fermentation→drying→primary dark tea. Conversely, press processing requires the following steps: primary dark tea→screening→ blending→steaming→piling fermentation→pressing→fungal fermentation→drying →FBT.
