# Supplementary figures and images for: Comparative Metagenomics Reveals Microbial Communities and Their Associated Functions in Two Types of Fuzhuan Brick Tea
Source: Front Microbiol. 2021 Sep 16;12:705681. doi: 10.3389/fmicb.2021.705681 (PMC8481837; doi:10.3389/fmicb.2021.705681)

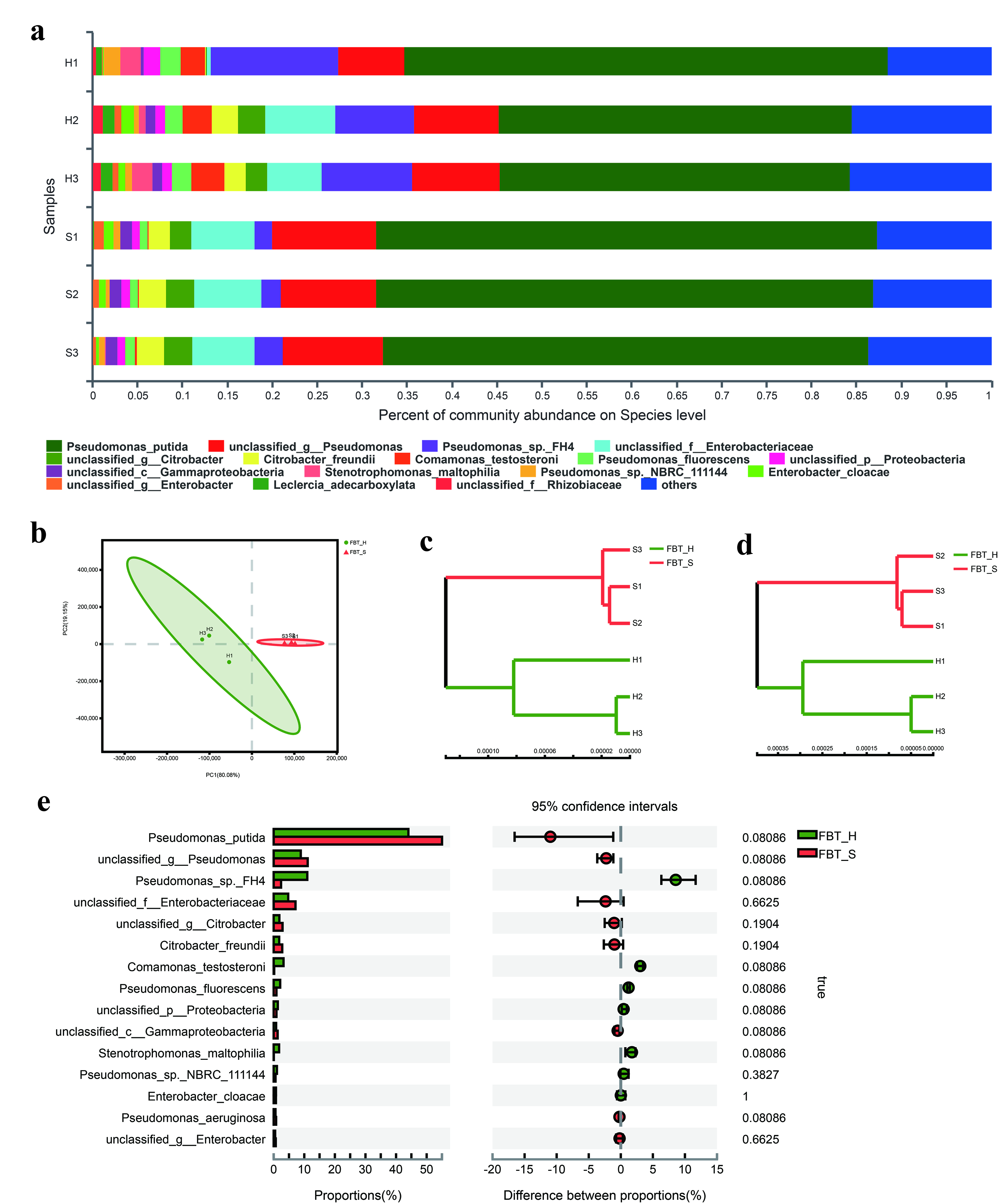

Supplement: Supplementary Figure 1 — Microbial community compositions among FBT samples. (A) Relative abundances of microbial community. (B) Variation in genus-level microbial composition within FBT samples. The proportion of variation explained by PCA1 and 2 was 80.08 and 19.15%, respectively. FBT_H: H1–H3. FBT_S: S1–S3. (C) Hierarchical clustering of genus-level taxonomic profiles. (D) Hierarchical clustering analysis of species-level profiles. (E) Comparison of species groups between FBT_H and FBT_S samples. [file Data_Sheet_1.ZIP › Figure S1.tif]

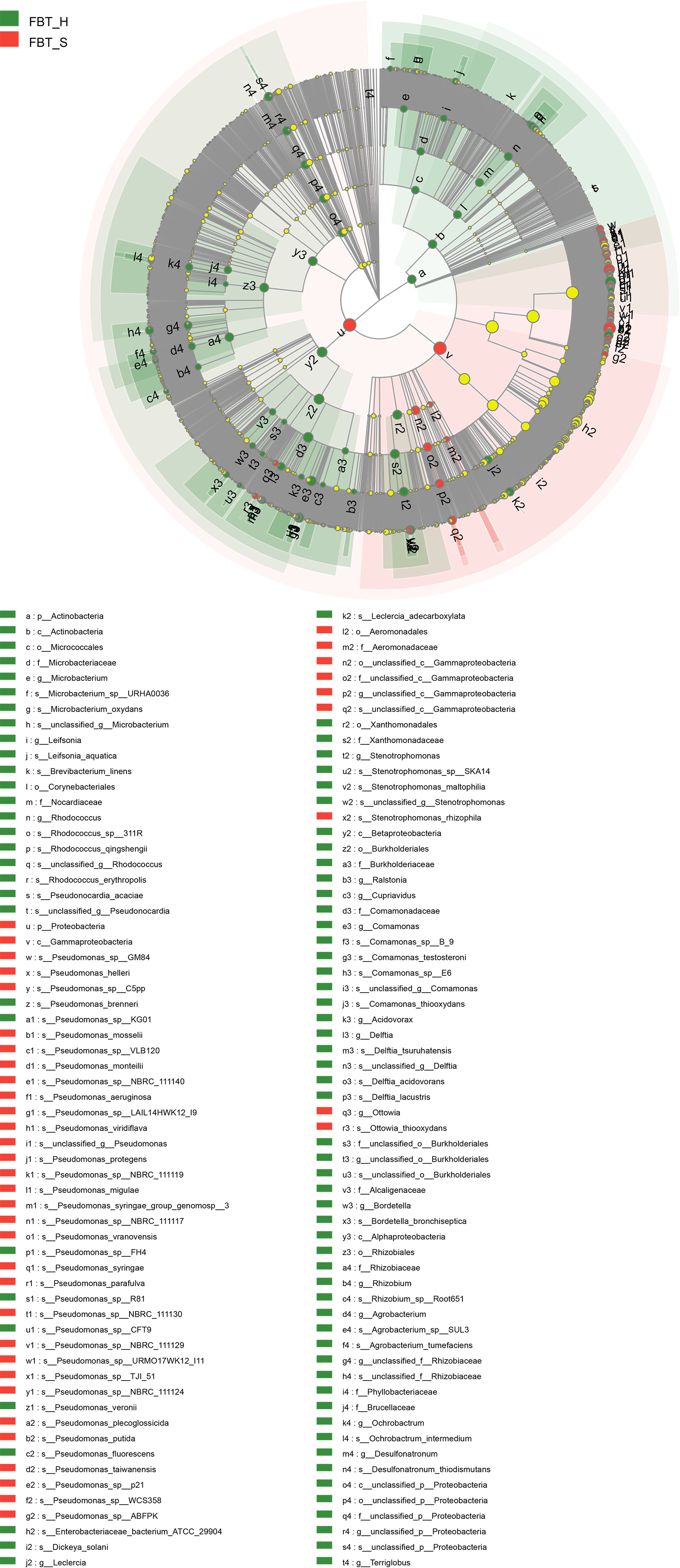

Supplement: Supplementary Figure 1 — Microbial community compositions among FBT samples. (A) Relative abundances of microbial community. (B) Variation in genus-level microbial composition within FBT samples. The proportion of variation explained by PCA1 and 2 was 80.08 and 19.15%, respectively. FBT_H: H1–H3. FBT_S: S1–S3. (C) Hierarchical clustering of genus-level taxonomic profiles. (D) Hierarchical clustering analysis of species-level profiles. (E) Comparison of species groups between FBT_H and FBT_S samples. [file Data_Sheet_1.ZIP › Figure S2.tif]

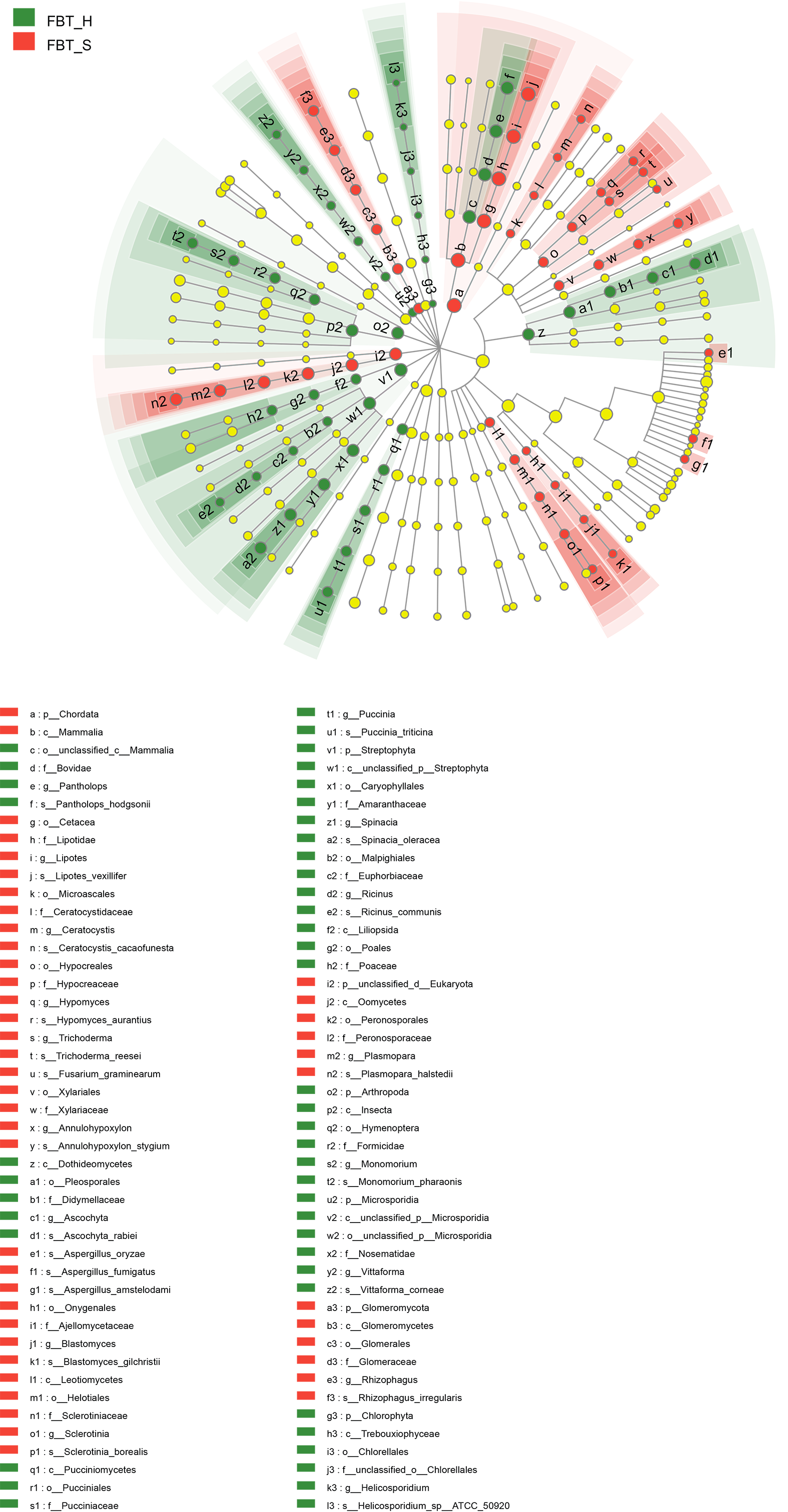

Supplement: Supplementary Figure 1 — Microbial community compositions among FBT samples. (A) Relative abundances of microbial community. (B) Variation in genus-level microbial composition within FBT samples. The proportion of variation explained by PCA1 and 2 was 80.08 and 19.15%, respectively. FBT_H: H1–H3. FBT_S: S1–S3. (C) Hierarchical clustering of genus-level taxonomic profiles. (D) Hierarchical clustering analysis of species-level profiles. (E) Comparison of species groups between FBT_H and FBT_S samples. [file Data_Sheet_1.ZIP › Figure S3.tif]

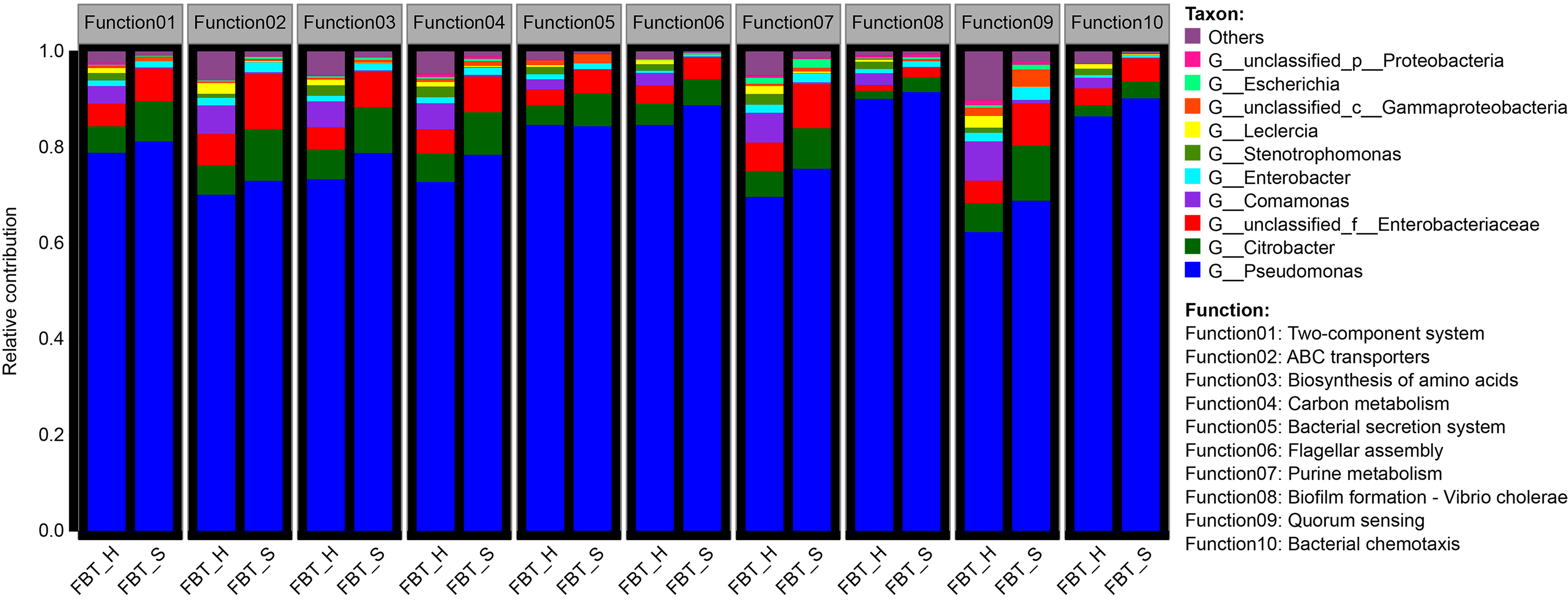

Supplement: Supplementary Figure 1 — Microbial community compositions among FBT samples. (A) Relative abundances of microbial community. (B) Variation in genus-level microbial composition within FBT samples. The proportion of variation explained by PCA1 and 2 was 80.08 and 19.15%, respectively. FBT_H: H1–H3. FBT_S: S1–S3. (C) Hierarchical clustering of genus-level taxonomic profiles. (D) Hierarchical clustering analysis of species-level profiles. (E) Comparison of species groups between FBT_H and FBT_S samples. [file Data_Sheet_1.ZIP › Figure S4.tif]

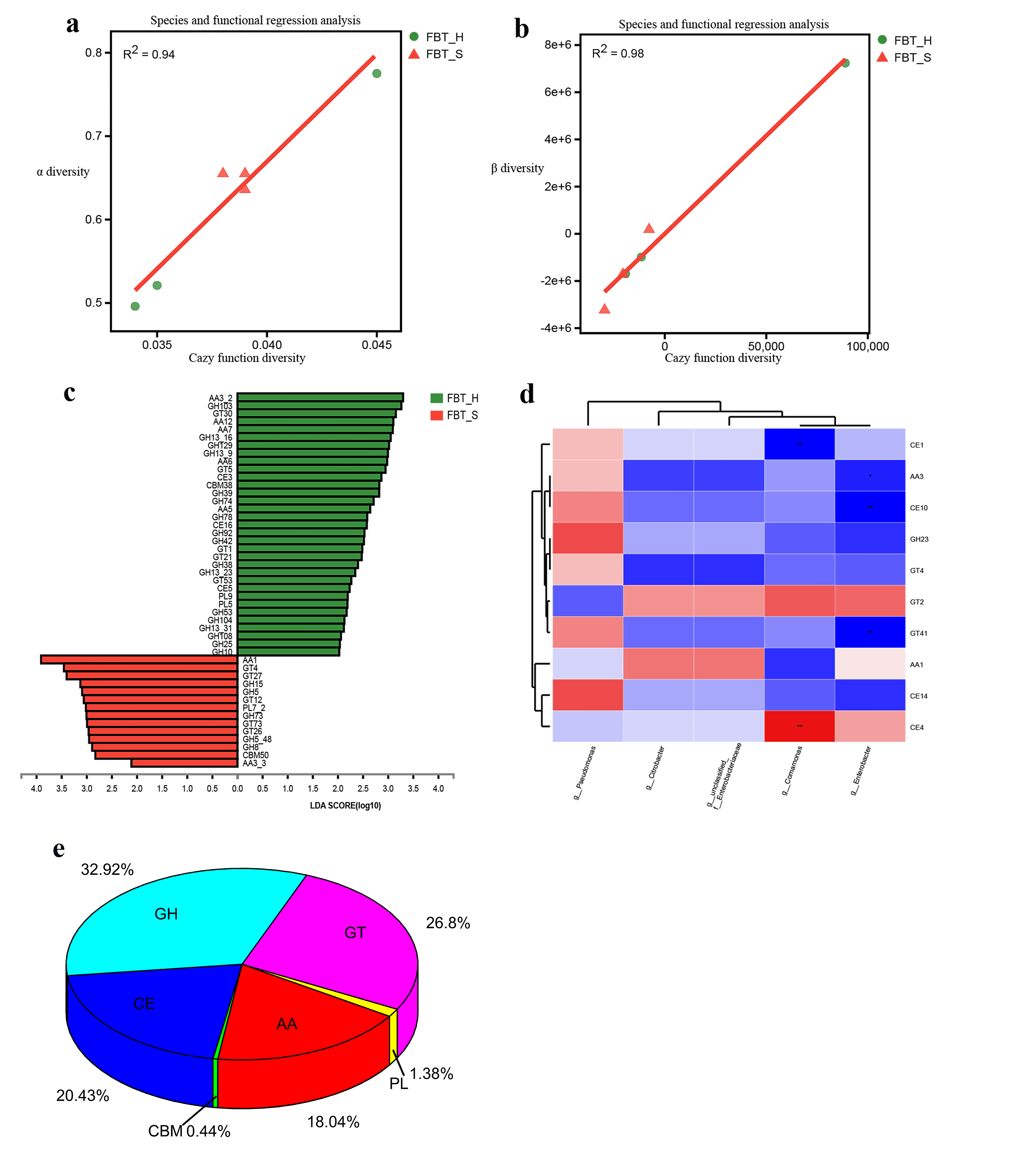

Supplement: Supplementary Figure 1 — Microbial community compositions among FBT samples. (A) Relative abundances of microbial community. (B) Variation in genus-level microbial composition within FBT samples. The proportion of variation explained by PCA1 and 2 was 80.08 and 19.15%, respectively. FBT_H: H1–H3. FBT_S: S1–S3. (C) Hierarchical clustering of genus-level taxonomic profiles. (D) Hierarchical clustering analysis of species-level profiles. (E) Comparison of species groups between FBT_H and FBT_S samples. [file Data_Sheet_1.ZIP › Figure S5.tif]

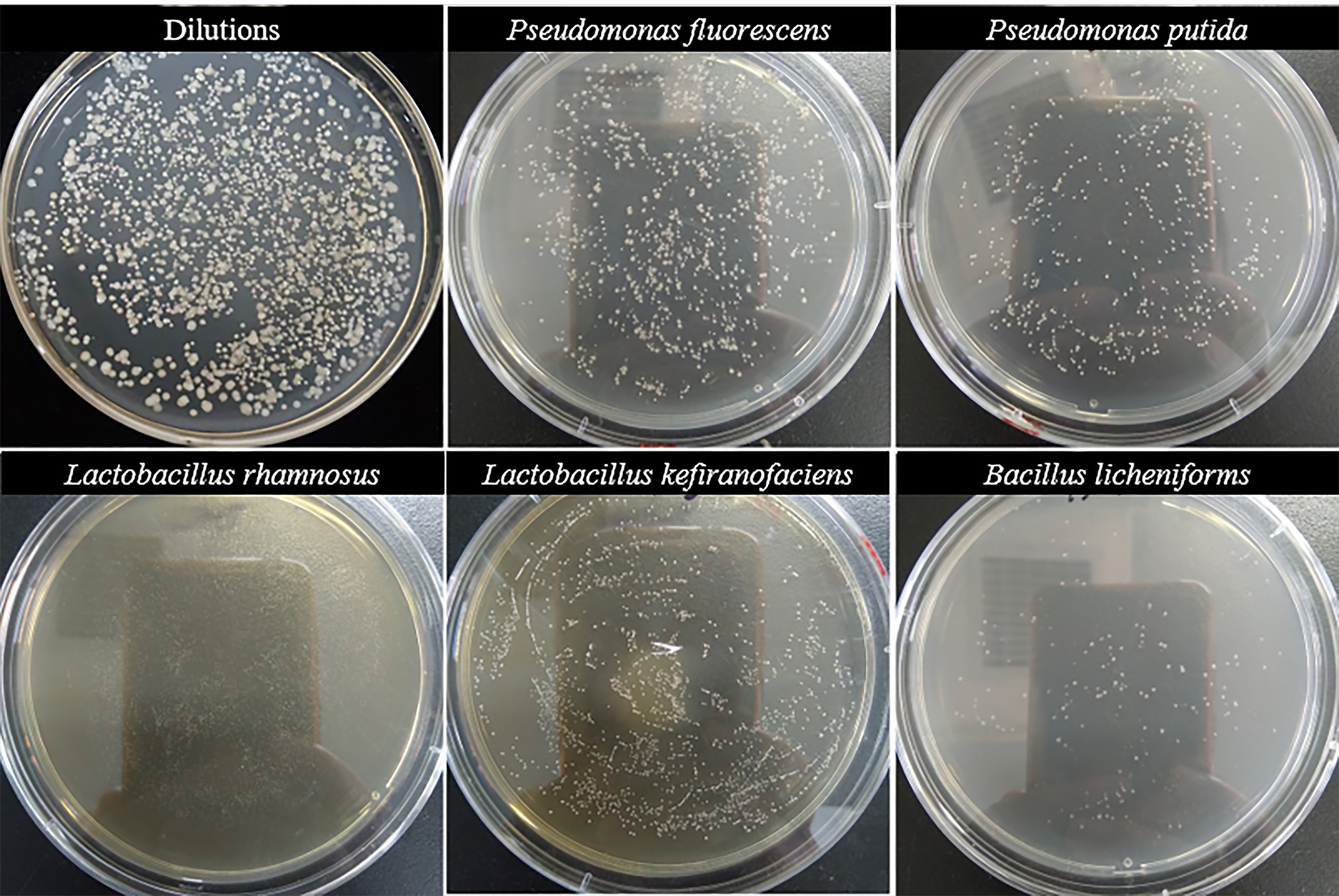

Supplement: Supplementary Figure 1 — Microbial community compositions among FBT samples. (A) Relative abundances of microbial community. (B) Variation in genus-level microbial composition within FBT samples. The proportion of variation explained by PCA1 and 2 was 80.08 and 19.15%, respectively. FBT_H: H1–H3. FBT_S: S1–S3. (C) Hierarchical clustering of genus-level taxonomic profiles. (D) Hierarchical clustering analysis of species-level profiles. (E) Comparison of species groups between FBT_H and FBT_S samples. [file Data_Sheet_1.ZIP › Figure S6.tif]
